# Management of a Thin Endometrium by Hysteroscopic Instillation of Platelet-Rich Plasma Into The Endomyometrial Junction: A Pilot Study

**DOI:** 10.3390/jcm9092795

**Published:** 2020-08-30

**Authors:** Meenu Agarwal, Liselotte Mettler, Smita Jain, Sandhya Meshram, Veronika Günther, Ibrahim Alkatout

**Affiliations:** 1 Morpheus Bliss Fertility Center, Sangamvadi, Pune, Maharashtra 411001, India; drmjainagarwal@hotmail.com (M.A.); smitakumari@gmail.com (S.J.); drsmbansode@gmail.com (S.M.); 2 Department of Obstetrics and Gynecology, Arnold-Heller-Str. 3, House C, University Hospitals Schleswig-Holstein, 24105 Kiel, Germany; profmettler@gmx.de (L.M.); veronika.guenther@uksh.de (V.G.)

**Keywords:** platelet-rich plasma (PRP), thin endometrium, hysteroscopy

## Abstract

In patients whose embryo transfer has been previously canceled due to a thin endometrium, the injection of platelet-rich plasma (PRP) guided by hysteroscopy into the endomyometrial junction improves endometrial thickness and vascularity. This may well serve as a novel approach for the management of these patients. In this study, 32 patients aged between 27 and 39 years, suffering from primary or secondary infertility, were selected for hysteroscopic instillation of PRP. This cross-sectional study included a retrospective assessment of the improvement of endometrial thickness (>7 mm) on the commencement of progesterone treatment in 24 of 32 patients (75%) after hysteroscopy-guided injections of PRP into the subendometrial zone. After PRP instillation, the endometrium was 7 mm or thicker in 24 of 32 patients, and all 24 patients underwent frozen embryo transfer. Moreover, 12 of 24 patients who underwent embryo transfer conceived, whereas 10 had a clinical pregnancy with visualization of cardiac activity at 6 weeks and two had a biochemical pregnancy. Our approach of PRP injection into the subendometrial region is consistent with the histologically proven regeneration of the endometrium from the endomyometrial junction. We observed an improvement of endometrial thickness and higher pregnancy rates in cases of previously canceled embryo transfer due to a thin endometrium.

## 1. Introduction

The optimal endometrial thickness for embryo transfer is assumed to be about 7 mm or more [1,2,3]. A thin endometrium has been identified as an important factor in implantation failure [4,5,6] because it is marked by high blood flow impedance of radial arteries of the uterine vasculature, poor epithelial growth, reduced expression of vascular endothelial growth factor (VEGF), and poor vascular development [7]. Various studies have shown the improvement of endometrial thickness with the use of prolonged estradiol valerate, aspirin, sildenafil citrate, L-arginine, and pentoxifylline, but no consensus has been achieved yet in this regard [8,9,10]. Intrauterine infusion of granulocyte colony-stimulating factor (G-CSF) is also not effective [11]. Some studies have shown better endometrial thickness (ET) after intrauterine platelet-rich plasma (PRP) [3], which prompted us to use this novel approach in patients who were unresponsive to the aforementioned modalities.

The uterus with its endometrium undergoes cyclical processes of regeneration, differentiation and shedding as part of the menstrual cycle [12]. The endometrium has an enormous ability to regenerate throughout the reproductive life: whether after births, curettage, or in menopausal women starting hormone replacement therapy, in most cases a proliferation of the endometrium under the influence of estrogen is observed. Stem or progenitor cells seem to be responsible for this regeneration process. The contribution of stem cells to endometrial regeneration was first described in 2004 [13,14]. Both progenitor cells within the endometrium and multipotent cells from bone marrow were shown to contribute to endometrial growth [15]. Evidence for the existence of adult stem and progenitor cells in human and mouse endometrium is becoming visible because functional stem cell assays are being applied to uterine cells and tissues [12]. CD140b+, CD146+, or SUSD2+ endometrial mesenchymal stem cells (eMSCs) and N-cadherin+ endometrial epithelial progenitor cells (eEPs) are just a few examples concerning types of stem/progenitor cells that have been identified [16].

Hysteroscopic instillation of PRP in the subendometrial region is a novel approach for the management of a refractory endometrium. PRP is autologous plasma derived from fresh whole blood enriched with platelets. It is prepared by collecting blood from peripheral veins and contains several growth factors such as VEGF, epidermal growth factor (EGF), platelet-derived growth factor (PDGF), transforming growth factor (TGF), and other cytokines that stimulate proliferation and growth [3,17]. In the endometrium, angiogenesis is a critical prerequisite for endometrial growth after menstruation and the achievement of a vascularized receptive endometrium for implantation [18,19,20]. A number of studies have shown that VEGF is expressed in the human endometrium and regulates vascularization at this site [7]. PRP, owing to its high content of growth factors, contributes to the improvement of endometrial thickness.

## 2. Materials and Methods

In this study, 32 women aged 27 to 39 years, suffering from primary or secondary infertility, were selected for hysteroscopic instillation of PRP at the Morpheus Bliss Fertility Center, Pune, India. There were three patients with one living child and secondary infertility, five patients with previous history of abortions, and 24 patients with primary infertility. The study was performed over a period of 14 months from July 2018 to September 2019. Day three transfers as well as blastocyst transfers were included. The objective of the study was to evaluate whether PRP injection into the endomyometrial junction, guided by hysteroscopy, improves endometrial thickness and vascularity in cases of previously canceled embryo transfer due to a thin endometrium. 

Endometrial and sub-endometrial blood flow was measured with a color doppler in the2D mode on a transvaginal scan using a GE Voluson S6 machine. Endometrial blood flow was detected by intra endometrial or the adjacent subendometrial region within 10 mm of echogenic endometrial borders. The patients where both endometrial and sub endometrial blood flow was detected after the instillation of PRP had an improved blood flow to the endometrium. Previous to the instillation of PRP, no visible endometrial/subendometrial blood flow was detected.

### 2.1. Inclusion Criteria

1. Patients in whom an embryo transfer had been canceled in previous cycles due to a thin endometrium (<7 mm), despite estrogen supplementation in increasing doses, intrauterine PRP instillation, intrauterine G-CSF, etc.

2. 7 patients in addition had PRP instilled intrauterine but did not show adequate improvement in the endometrial thickness; hence, embryo transfer was cancelled. 

3. 13 patients in addition to estrogen supplementation had G-CSF instilled intrauterine but did not show adequate improvement in the endometrial thickness; hence, embryo transfer was cancelled.

4. Patients undergoing frozen embryo transfer cycles.

5. Women with a normal transvaginal ultrasound and no evidence of a clinically significant abnormality in the uterus or adnexa.

6. Negative acid fast bacillus (AFB) culture for genital tuberculosis. 

The primary outcome was defined as an endometrial thickness of >7 mm on commencement of progesterone, resulting in an embryo transfer. The secondary outcome was a positive beta-hCG level and clinical proof of pregnancy.

Autologous platelet-rich plasma (A-PRP) is a preparation that contains a high concentration of platelet growth factors, well above normal levels in blood. A-PRP is developed from autologous blood and is therefore inherently safe and free of transmissible diseases, such as HIV or hepatitis. The concentration of platelets in PRP delivers a large number of growth factors in biologically determined ratios, which distinguishes this substance from recombinant growth factor. PRP contains platelet and growth factor in levels ranging from 80% to 98%, and has been extensively used to improve tissue repair and hair growth.

### 2.2. Methods for PRP Preparation 

Eight mL of the patient’s blood was taken in a PRP tube (GeoPRP kit -US-FDA approved regenlab PRP Kit). The tube was shaken thoroughly and the contents were centrifuged at 3600 rpm for 6 min. The tube was then shaken upside down 20 times for homogenization, and the supernatant PRP collected with an 18 G needle.

### 2.3. PRP Injection

**Figure 1:**
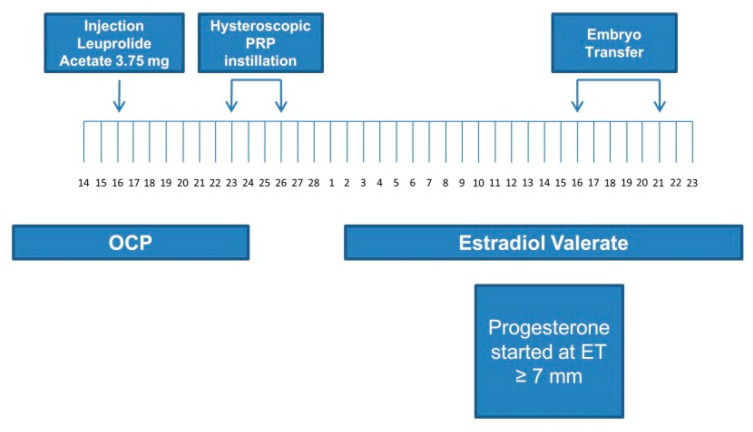
Preparation and procedure of the platelet-rich plasma instillation. OCP: oral contraceptive pills; PRP: platelet-rich plasma; ET: endometrium thickness.

All patients were instructed to take oral contraceptive pills (OCPs) once daily for 21 days in a previous cycle. Next, 3.75 mg of leuprolide acetate was administered by the intramuscular route to all patients on day 16 of OCP intake for down-regulation of gonadotropins (Figure 1).

All patients underwent hysteroscopic instillation of PRP in the subendometrial region 7–10 days after the injection of leuprolide (Figure 1).

**Figure 2:**
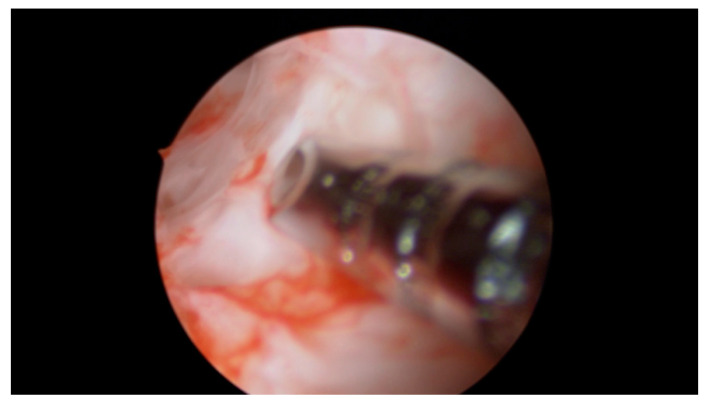
Hysteroscopic instillation of PRP, the beveled edge of the ovum. pick up needle is oriented towards the uterine cavity in order to determine the exact depth of insertion.

**Figure 3:**
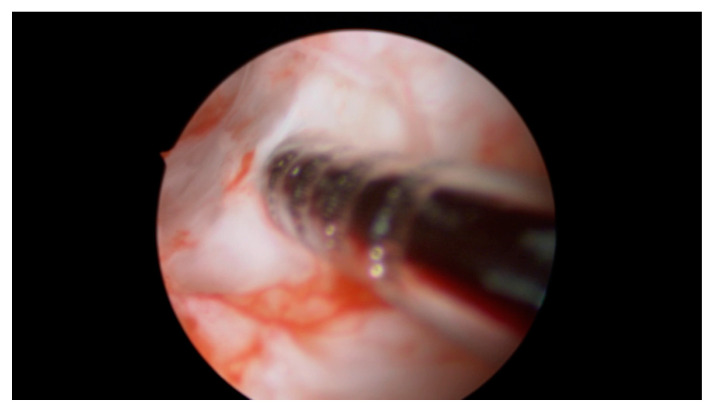
The markings on the ovum pickup needle help to determine the correct depth of insertion.

**Figure 4:**
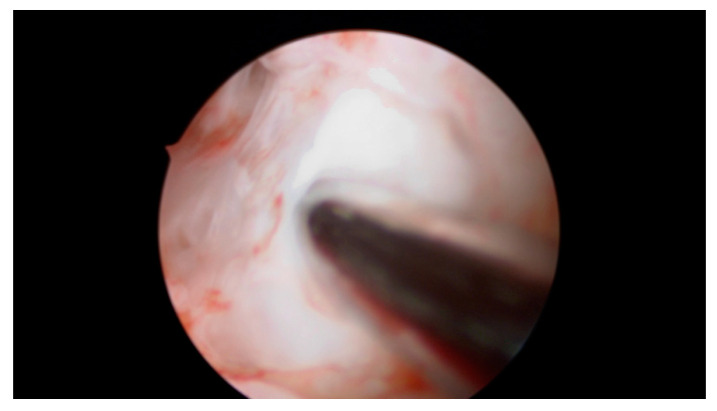
Prepared PRP is pushed into the endomyometrial junction and the needle is withdrawn under vision. No leakage of injected fluid was seen on withdrawal of the needle; PRP was instilled within 20 to 30 min of its preparation.

A total of 4 mL of PRP was injected with an ovum pickup needle into the subendometrial region in all four walls of the cavity (1.0 mL in each wall) under hysteroscopic guidance. Optimum instillation was ensured by keeping the beveled edge of the ovum pickup (OPU) needle facing the cavity in slanting position (Figure 2, Figure 3 and Figure 4).

### 2.4. Endometrial Preparation and Embryo Transfer

One week after cessation of the OCP, on the second day of menses (withdrawal bleeding) in the embryo transfer cycle, women were given estradiol valerate tablets at a dose of 6 mg; the dose was progressively increased to 12 mg per day. From day 6 onward, a transvaginal ultrasonography was performed to measure endometrial thickness on alternate days. To assess ET, the same examiner measured the thickest portion in the longitudinal axis of the uterus. Luteal phase support with 400 mg of vaginal progesterone was started when the ET reached the optimum thickness of 7 mm. Frozen-thawed embryo transfer was performed after synchronizing the day of progesterone treatment with the age of the embryo (only day 3 or day 5 embryos were transferred). Both estradiol valerate and progesterone were continued in the same dosage for luteal support. Serum beta-hCG levels were measured two weeks after embryo transfer. A transvaginal sonography was performed two weeks later in patients with positive beta-hCG levels in order to confirm a clinical pregnancy.

## 3. Results

The instillation of PRP caused no side effects and was well tolerated by all patients. In the subsequent cycle monitored to day 15, ET was 7 mm or thicker in 24 of 32 patients, and 6-7 mm in 4 of 32 patients. Endometrial thickness did not improve in four of 32 patients and remained below 6 mm (Table 1).

Progesterone was started the day the endometrium achieved a thickness of 7 mm or more. Subendometrial blood flow increased significantly in 28 of 32 patients. The mean increase in ET was 1.5 to 2 mm. Next, 24 patients underwent frozen embryo transfer. In 8 patients (25%), the cycles were canceled because they did not achieve optimal endometrial thickness. 

Moreover, 12 patients had day 3 embryos transferred and 12 patients had two blastocysts transferred on day 5. Luteal phase support was given to all 24 patients. Further, of the 12 of 24 patients who underwent embryo transfer conceived, 10 had a clinical pregnancy with visualization of cardiac activity at 6 weeks, and two had a biochemical pregnancy. In 8 of 10 pregnant patients, the pregnancy progressed uneventfully. 

Two patients had a missed abortion in the first trimester. Five patients have already delivered and three pregnancies are in progress at the date of this publication (Table 2).

## 4. Discussion

The clinical application of platelet-rich plasma (PRP) has increased markedly in the last decade. PRP has been extensively used for hair regeneration and tissue regeneration in cosmetic dermatology and gynecology. 

PRP is an autologous blood plasma enriched with four- to five-fold higher levels of platelets than those in circulating blood. PRP stimulates proliferation and regeneration with a large quantity of growth factors and cytokines, including PDGF, TGF, VEGF, EGF, fibroblast growth factor (FGF), insulin-like growth factor I, II (IGF I, II), interleukin 8 (IL-8), and connective tissue growth factor (CTGF) [3]. 

We analyzed hysteroscopic instillation of PRP in the endomyometrial junction to improve endometrial thickness for embryo transfer in the subsequent cycle. As such, 75% of the patients achieved an endometrial thickness of 7 mm or more, underwent an embryo transfer, and 50% of the patients conceived.

Considering these results, the question arises as to what the possible causes of non-proliferating endometrium might be. Herein, 25% of the patients did not achieve an endometrial thickness of 7 mm or more. Anatomical or structural abnormalities such as Asherman’s syndrome, for example, could be the cause of a lack of endometrial proliferation. Intrauterine adhesions with symptoms like hypomenorrhea/amenorrhea, reduced fertility, or abnormal placentation are known under the term Asherman’s syndrome. Possible causes might be a lesion of the basal layer of the endometrium (i.e., after curettage), hysteroscopic surgery, or uterine artery embolization [21]. Furthermore, unspecific factors can be discussed, like age, race, nutritional status, and previous infections. Nonetheless, a history of trauma seems to be the determining factor [22]. 

Another possible cause of the less proliferated endometrium could be the lack of special marker molecules, which are considered characteristic for endometrial receptivity. A German study group [23] analyzed Leukemia inhibitory factor (LIF), vascular endothelial growth factor (VEGF), and β 3 integrin, which are marker molecules for endometrial receptivity. These marker molecules were found to be inadequately expressed or completely absent in the endometrial tissue samples the specific group of subfertile patients with suspected endometrial deficiency [23]. 

The human endometrium has its own immunosensititvity to sex steroid hormones. There are different endometrial concentrations of estrogen and progesterone receptors throughout the menstrual cycle [24]. The molecular and cellular events mediating these changes are not fully understood. The establishment of normal endometrial receptivity appears to be tightly associated with the down-regulation of epithelial progesterone receptor [24,25]. A histological analysis of endometrial tissue can be done in order evaluate the estrogen receptor concentrations. Low estrogen receptor concentrations seem to be related to low pregnancy rates [26]. 

Maekawa et al. [27] found an aberrant Th1-pro-inflammatory/Th2-anti-inflammatory balance and increased cytotoxic condition in patients with thin endometrium. Genome-wide mRNA expression analysis was used in order to show the different expression profiles that exist in case of patients with thin endometrium compared to the control group with an endometrium ≥7 mm [27]. An overactivation of the uNK cells and a cytotoxic/Th1 pro-inflammatory environment was found to be present in a thin endometrium, which is associated with implantation failure [27]. Possible limitations of our study are based on previously cited studies. We have not examined our patients more closely for possible causes of thin endometrium and we did not investigate marker molecules for endometrial receptivity, nor did we perform an mRNA expression analysis. 

A further point of criticism is that the number of cases with 32 patients is expandable and no subgroup analysis was performed with regard to primary and secondary sterility, habitual miscarriage, and recurrent implantation failure. This should be considered and integrated in further studies. 

To further prove the benefit of the application method (instillation vs. infusion of PRP) the following setting would be desirable in a further study: the case group would receive a hysteroscopic instillation of the PRP, while the control group would receive an infusion of the PRP into the uterine cavity.

In our study, there were three patients with one living child and secondary infertility, five patients with previous history of abortions, and 24 patients with primary infertility. 

Infertility is defined as failure to achieve clinical pregnancy after at least 12 months of unprotected coitus. Couples who already have a child but waited another 12 months or more for a new pregnancy suffer from secondary infertility also fall under this description.

The causes of primary infertility can be many and varied. Endometriosis, uterine abnormality—such as septa, polyps, and fibroids—chromosomal aberrations, infections, obesity, thrombophilia, or immunological causes are a few examples. An essential difference in the pathomechanism of primary versus secondary infertility is the age of the woman. The age for the first maternity has already shifted significantly backwards in today’s society. In this case, a reduced egg cell reserve and a limited quality of the oocytes can be assumed [28]. 

Previous traumas affecting the endometrium can promote or even cause secondary infertility. Possible reasons might be caesarean section, abrasion, or hysteroscopic surgery. These procedures can impede adequate proliferation of the endometrium and in rare cases cause Asherman’s syndrome [21]. 

Over time, other gynecological conditions that do not cause much discomfort at an earlier age can also become real obstacles when it comes to conception. These include, for example, increased symptoms of endometriosis, fibroids of the uterus, intracavitary polyps, or increasing disorders of ovulation. Other clinical pictures that make a new pregnancy difficult and that increase over the years include rheumatological and degenerative diseases, high blood pressure and diabetes.

Furthermore, male fertility disorders can be the trigger for secondary sterility. In men with advanced age, reduced sperm quality (concerning the quantity and motility of the sperm cells), erection problems, high blood pressure, diabetes, and other impairments can become increasingly apparent [29].

Since the first in vitro fertilization (IVF) attempts in the mid-1970s, a number of assisted reproduction technologies (ART) have been used. These include stimulation protocols, embryo culture/culture medium, and embryonic growth to the blastocyst stage. The human endometrium undergoes significant changes during implantation. Immune cells and their secretions, such as granulocyte colony-stimulating factor (G-CSF) in the luteal phase, play an important role in the process of implantation. 

In contrast to the approaches mentioned above, the phenomenon of implantation itself is not fully understood. Little is known about the mother-fetus dialog and the individual steps or possible sources of error in apposition, adhesion, and invasion. This has resulted in limited options for achieving implantation. One of the options is to influence the maternal immune system in order to promote implantation and the continuation of pregnancy. Due to heterogeneous data regarding live birth rates, immunomodulatory therapies, such as intralipid infusion, immunization with partner lymphocytes, or glucocorticoid administration, are not included in the corresponding guidelines [30].

PRP is a relatively new method of enhancing endometrial thickness and achieving higher pregnancy and live birth rates. 

Chang et al. published the first trial on the use of PRP in human reproduction technologies in 2015. They showed the efficacy of intrauterine infusion of PRP for endometrial growth in women with a thin endometrium. All five treated patients became pregnant and delivered their infants at term; the fifth patient had an abortion due to an XO fetus [17]. In contrast to our study, Chang et al. infused PRP into the uterine cavity with a catheter, and the embryo was transferred during the same cycle. Endometrial thickness was re-assessed 72 h after infusion. The infusion had to be repeated in four patients due to inadequate endometrial thickness.

A pilot study by Zadehmodarres et al. included 10 patients with a history of inadequate endometrial growth into the study. The patients got infusion of PRP before frozen-thawed embryo transfer. In all patients, endometrial thickness increased after PRP and embryo transfer was done in all of them. Five patients were pregnant. According to this study, it seems that PRP was effective for endometrial growth in patient with thin endometrium [3]. 

Our study has added a novelty to this and already new method: the hysteroscopically controlled instillation of PRP. This approach has not yet been described in the literature and is therefore an absolute novelty. The idea is not only to perform an infusion or irrigation but to inject PRP directly into the stem/progenitor cell site into the junctional zone to have even more influence on angiogenetic and growth cells. This method was performed in patients with thin endometrium, who had cycle cancellation due to thin endometrium despite of intrauterine PRP or GCF infusion before. 

A study group from Teheran [31] investigated 138 patients with repeated implantation failure in 2016 and 2017. The women failed to conceive after three or more embryo transfers with high-quality embryos. The intrauterine PRP infusion was performed 48 h before blastocyst transfer. A control group received standard treatment, while 97 patients in the study group were given PRP infusions. The biochemical pregnancy rate was higher in the PRP group than in controls (53.06% versus 27.08%, respectively; *p* = 0.009) and the clinical pregnancy rate was also higher in the PRP group than in controls (44.89% versus 16.66%, respectively; *p* = 0.003). The authors concluded that PRP infusions cause higher pregnancy rates in women with repeated implantation failure [31]. 

In contrast to our study, the above mentioned patients suffered from repeated implantation failure, whose causes were not clearly specified. Implantation failure was not exclusively due to a subliminally proliferated endometrium. Instead of using a catheter, we instilled PRP into the endomyometrial junction under hysteroscopic guidance. Notably, the patients benefited from the treatment in both studies. 

The above mentioned study group performed a randomized double-blind controlled trial concerning PRP [32]. In total, 60 patients who had a history of canceled frozen-thawed embryo transfer cycle due to a thin endometrium (<7 mm) were randomly assigned to PRP or a sham-catheter group in a double-blind manner. Intrauterine PRP infusions or a sham-catheter infusion was performed on day 11-12 and was repeated after 48 h, if necessary. All participants needed a second intervention because of inadequate endometrial expansion. After the second intervention, the endometrial thickness was 7.21 ± 0.18 and 5.76 ± 0.97 mm in the PRP group and sham-catheter group, respectively; the difference was significant (*p* < 0.001). Embryo transfer was performed in all patients in the PRP group and just six women in the sham-catheter group. A chemical pregnancy was reported in 12 cases in the PRP group and two cases in the sham-catheter group [32].

Maleki-Hajiagha et al. [33] performed a systematic review and meta-analysis concerning PRP; seven studies encompassing 625 patients (311 cases and 314 controls) were included. The probability of chemical pregnancy, clinical pregnancy, and implantation rates were significantly higher (*p* < 0.001) in women who received PRP compared to controls. The two groups did not differ in regard to miscarriages. Following the intervention, endometrial thickness increased in women who received PRP, but did not increase in the controls. The findings of this systematic review suggest that PRP is an alternative treatment strategy in patients with a thin endometrium and recurrent implantation failure (RIF) [33]. 

In our pilot study, we examined the new aspect of the injection site for the first time. In contrast to the “simple” infusion of platelet rich plasma into the uterine cavity, we injected the PRP under hysteroscopic view in the endomyometrial junction. Although reported as a safe procedure, it would be interesting to evaluate the long-term consequences. Indeed, the pathogenesis of endometriosis and adenomyosis may involve micro trauma at the junctional zone. It would be the task of the following studies, should the hysteroscopic instillation of PRP be used regularly, to investigate whether there is an association between a higher incidence of adenomyosis after prior PRP infiltration. Adenomyosis is associated with a higher risk of infertility. In this case, it would have to be considered whether the benefit of PRP outweighs the risk of possibly indicated adenomyosis.

The origin and pathogenesis of endometriosis is not fully understood. A possible developmental mechanism for endometriosis might be the dysregulation of endometrial stem cells, maybe in combination with the Sampson theory of retrograde menstruation [34,35]. When progenitor cells are shed at the time of menstruation, they can implant and generate endometrium in ectopic locations, for example, in the small pelvis or in the myometrium. The celomic theory describes that embryonic cells from the Müllerian ducts persist in ectopic locations. At puberty, stimulated by estrogens, they grow to build up endometriotic lesions [36]. Nyholt et al. [37] described in their meta-analysis five novel loci related to the risk of developing endometriosis. All five are involved in sex steroid pathways. Furthermore, there is evidence that endometriosis is a pelvic inflammatory condition with a peritoneal fluid showing an increased concentration of activated macrophages and cytokines There are novel insights concerning pathogenesis of adenomyosis. Ibrahim et al. [38] described so called pale cells, a cell population found among the epithelial cells of the basal glands at the endomyometrial-junctional-zone. The cells got their name because of the electro-lucent cytoplasm and seem to migrate into the stroma of the basal endometrium and subsequently into the myometrium. Those pale cells have also been observed in the pelvic peritoneal endometriotic lesions, irrespective of the cycle day [38]. Going back to our study, one could consider that microtrauma in the area of the junctional zone to promote the migration of the pale cells and thus the development of adenomyosis. The same research group showed that the presence of myofibroblasts at the junctional zone is microscopic evidence of chronic tissue trauma in patients with adenomyosis. They are of nonmyometrial origin, as they lack desmin immunolabeling [39]. Further studies are necessary to investigate the influence of PRP instillation into the junctional zone on possible microtrauma and thus endometriosis development. 

Nevertheless, many of the current studies concerning PRP comprise small numbers of cases and have different study designs. Before PRP can be recommended in clinical routine, it is necessary to perform further large prospective randomized controlled trials (RCTs) of high quality and identify women who would benefit most from PRP [40].

PRP is a new option for the improvement the endometrial thickness in women with a thin endometrium; its use is considered safe because it is derived from the patient’s own blood.

Our approach of injecting PRP into the subendometrial region is consistent with the histologically proven regeneration of the endometrium from the endomyometrial junction. Instillation of PRP a few weeks before embryo transfer rather than intrauterine instillation on day 10 or 12 of same cycle ensures the maximum benefit for the patient. In many previous studies, PRP infusion had to be repeated because of no change in endometrial thickness [32,41]

Despite the use of PRP for musculoskeletal injuries, dentistry, and other medical fields including human-assisted reproduction, the method of preparing PRP for clinical use is far from standardized [42]. Furthermore, the limited body of clinical data on the subject is largely derived from non-randomized trials. We need well-designed randomized studies and basic research at the cellular and molecular level to improve our understanding of PRP as well as determine specific clinical situations for its use. 

## 5. Conclusions

The thin endometrium has perplexed ART clinicians worldwide and still is a challenge in terms of treatment. The method of improving endometrial thickness by hysteroscopic instillation of PRP, developed at our center, yielded promising results and has created new options for the use of PRP in infertile women with previously canceled cycles due to a thin endometrium.

## Figures and Tables

**Table 1:** Endometrial thickness following platelet rich plasma (PRP) administration.

Endometrial Thickness	Number of Patients
≥7 mm (on the day of progesterone start and embryo transfer done)	24 (75%)
6–7 mm (embryo transfer not done)	4 (12.5%)
<6 mm no improvement (embryo transfer not done)	4 (12.5%)

**Table 2:** Pregnancy outcome in 24 patients who underwent frozen embryo transfer after platelet-rich plasma (PRP) instillation.

Pregnancy Outcome	Number of Patients (24 in Total)
Beta-hCG positive	12 (50%)
Clinical pregnancy	10 (41.66%)
Biochemical pregnancy	2 (8.33%)
Ongoing pregnancy	3 (12.5%)
Live birth	5 (20.83%)
Missed abortion	2 (8.33%)
